# Navigating uncharted waters: select practical considerations in radiology AI compliance with the EU AI Act

**DOI:** 10.1038/s41746-025-02094-z

**Published:** 2025-10-25

**Authors:** Jaka Potočnik, Damjan Fujs

**Affiliations:** 1 School of Medicine, University College Dublin, Dublin, Ireland; 2 Faculty of Computer and Information Science, University of Ljubljana, Ljubljana, Slovenia

**Keywords:** Health policy, Policy

## Abstract

Artificial intelligence (AI) is transforming traditional medicine, particularly in radiology. Its integration across patient care stages has made it increasingly ubiquitous. The European Union’s (EU) AI Act will additionally regulate AI-enabled solutions within the EU. However, without standardized guidelines, the Act’s flexibility poses practical challenges for providers and deployers, leading to inconsistencies in meeting requirements for high-risk systems like radiology AI, potentially impacting patients’ fundamental rights and safety.

Many healthcare providers in the (EU) have already integrated AI-enabled medical devices or systems into their radiology workflows. While their conformity with the Medical Device Regulation (MDR)^1^ was primarily assessed by a notified body, all of these will fall under the scope of the AI Act^2^. This new EU regulation complements the existing MDR and has been fully in force since August 2025 for general-purpose AI and foundation models, with a transition period of up to two years for high-risk AI systems, such as medical devices. Most applications of AI in radiology are likely to be classified as high-risk due to processing personal health data, influencing human experts’ decision-making, impacting patient outcomes and quality of life. As such, these systems must uphold the rights specified in the Charter^3^, including the right to protection of personal data (Article 8), non-discrimination (Article 21), equality between men and women (Article 23), and access to health care (Article 35). Consequently, more stringent requirements apply to high-risk AI systems^4^.

## Risk management system

The AI Act mandates a risk management system (RMS) for high-risk AI systems under Article 9, building on existing MDR risk management requirements. An RMS is essential for identifying, assessing, and mitigating risks in high-risk AI systems during development and immediately upon deployment. However, the Act provides limited guidance on designing these systems or identifying known, reasonably foreseeable, and less likely risks through risk analysis methods. As a result, providers and deployers may design RMS that vary in structure and content, even for AI systems with the same purpose and underlying technicalities. For example, some providers may align their RMS with ISO 14971^5^, while others may adopt a simplified, ad-hoc RMS relying on internal checklists. Just this variability can lead to inconsistencies in risk analysis, identification, and evaluation. Moreover, different mitigation measures for identical risks may differ in effectiveness. Inconsistent metrics, thresholds for defining risks, and personnel responsibilities further exacerbate variability. For radiology AI, input from a multidisciplinary team is essential to identify as many risks—known and reasonably foreseeable—as possible, to ensure a robust RMS. Certainly, similar AI systems, considering their design and/or intended task(s), will have some risks and mitigation measures in common. Although the AI Act’s RMS requirements are intentionally broad to accommodate diverse AI applications, this lack of specificity can complicate implementation and negatively impact patients’ fundamental rights and safety.

## Data and data governance

In Article 10, the Act stipulates that datasets used to train, validate, and test high-risk AI systems must be sufficiently representative and complete for the intended purpose. A plethora of peer-reviewed studies focused on the development of radiology AI exist. The vast majority utilized limited, non-representative datasets that may introduce different types of bias. In fact, there appears to be insufficient effort to create systems for real-world use. Most papers fail to provide raw anonymised data, detailed dataset descriptions, or methodologies for quantifying and evaluating dataset representativeness and completeness [Review ref. ^6^]. The outputs of such studies typically demonstrate feasibility, although in limited contexts, but fail to meet new regulatory requirements. Similarly, vendors offering commercial AI solutions often lack transparency and fail to disclose sufficient dataset or technical details^7^. Such practices will no longer be acceptable under Article 13 of the AI Act, requiring greater transparency and the provision of detailed information to deployers. This will allow deployers to interpret the results of a system.

How can one determine if a dataset is representative of a specific population and complete, and how can this be done quantitatively and confidently? The updated Checklist for Artificial Intelligence in Medical Imaging^8^, which promotes transparency and reproducibility, does not provide clear guidance on this ambiguity. Instead, it expects that authors ‘describe how well the data align with the intended use and target population of the model’, which circles back to the initial question. For instance, imagine we aim to build an AI system to detect lung cancer on chest radiographs for the busiest public hospital in Berlin, the largest capital city in the EU.

Starting with data representativeness, Berlin’s demographic diversity must be considered. Its multicultural population, including immigrant communities, requires the inclusion of diverse ethnic groups to avoid selection bias. Additionally, our training dataset should reflect varied socioeconomic backgrounds and environmental exposures, as access to healthcare, smoking, and urban air pollution can influence disease presentation. In other words, geographic and environmental relevance are critical in this context. To be thorough, intra-variability of individual features should not be ignored; for example, whether a patient smokes one cigarette or one pack per day should be taken into account, and each patient group in relation to this feature should be adequately represented. The disease spectrum is also a key feature of the dataset; it should include different cancer subtypes, stages, and comorbidities specific to Berlin’s patient population. Not to mention that the dataset should accurately reflect realistic prevalence rates.

Continuing with data completeness, a large training dataset is essential for developing a robust model. Expert data annotations, preferably with consensus from multiple board-certified radiologists, are necessary. Annotations should extend beyond confirming diagnoses to include details on lesion location, size, type, etc. Smoking history, symptoms, family health history, and other clinical metadata are equally critical. If available, temporal and longitudinal data should be included to capture disease progression, treatment response, or remission. Additionally, the dataset must account for operator-to-operator variability, differences in radiographic image quality, and variations across X-ray machines. Image artifacts—such as hair, jewelry, motion blur, overlapping structures, skin fold—can obscure or mimic certain pathologies and must be represented in the dataset.

Many variables and their intra-variability influence data representativeness and completeness, and these are highly context dependent. Currently, there is no standardized method to quantify dataset representativeness and completeness for radiology AI across different contexts, leaving a gray area in interpreting and addressing these critical attributes that impact fundamental rights. Ultimately, if either attribute is inadequately addressed, certain patient groups may be underdiagnosed or denied equitable access to healthcare by the AI system. Moreover, in addition to the standardization of the data resources, there is also a need for standardization of other regulatory elements such as algorithm transparency, risk management, data security and harmonization of regulatory frameworks (i.e., to have a single and general framework/standard)^9^, as the leading countries are currently going their own way (regulatory/standardization differences between the United States, The EU, Australia, China, etc.).

## Post-market monitoring

The performance and safety of high-risk AI systems must be continuously monitored upon their deployment; thus, providers need to design a post-market monitoring (PMM) plan and execute it through continuous monitoring of the device throughout its lifecycle (Article 72). It is important to note that both RMS and data governance form part of a PMM system. These should be tailored to the design and purpose of individual AI systems. Its efficacy and efficiency highly depend on the content of the RMS and attention devoted to specific characteristics of datasets in different contexts and their processing. Yet, what constitutes a good, comprehensive PMM system—whether standalone or integrated, with an intuitive user interface and informative dashboard, continuously or periodically communicating with the AI system—remains unclear. The practical issues discussed are already evident in real-world settings. In the Netherlands, the Health and Youth Care Inspectorate (IGJ) identified insufficient and inconsistent PMM at all 13 medical device providers visited in 2023 and 2024^10^. The Authority raised several concerns, including poorly developed PMM plans, partial implementation of PMM, providers’ limited knowledge and skills regarding PMM, overly broad PMM scopes lacking customization for specific medical device types, and more. Nearly half of the providers did not have a PMM plan at all. For some with a PMM plan, the plans were grossly incomplete. Similarly, Swissmedic inspected 27 medical device manufacturers in Switzerland, of which 19 (70%) failed to provide adequate PMM documentation^11^. Both IGJ and Swissmedic conducted inspections of manufacturers producing Class I (low-risk) medical devices. When providers do not give priority and appropriate attention to PMM planning and execution, they risk harming patient safety and breaching fundamental rights. At the time of writing this commentary, regulatory evidence of compliance for manufacturers of Class III (high-risk) medical devices in the EU is unavailable.

Challenges in harmonizing a PMM system’s design due to varying interpretations and limited guidance are anticipated for high-risk AI systems in radiology. Given the fact that many public datasets are limited, in terms of geographic, demographic, genetic, and epidemiologic inclusion, radiology AI can underperform in various patient subgroups^12^. How will the differences between the training data and that presented to the AI system in real clinical scenarios be monitored? If there is a mismatch between the conditions for model training and clinical use, data drift should be flagged by the PMM system as soon as the difference is statistically significant. This means that individual dataset features and their intra-variability need to be measured. Any concept drift, where the relationship between input data and target features changes over time, should also not remain undetected^13^. Deployers of radiology AI will need to remain vigilant about the model’s performance during routine use and potentially provide new target labels in real time to enable realistic performance evaluation and potential model retraining. It is well-known that the clinical performance of AI systems is lower, compared to the testing accuracy on hold out sets reported by vendors. Disparity testing may play an important role in determining realistic performance across various patient subgroups, as many AI-enabled studies have issues with moderate or high disparity^14,15^. Regulatory bodies have not reached a definitive conclusion on how PMM should work in practice. No notified body has yet been appointed to audit against the AI Act.

In summary, this commentary highlights practical concerns related to achieving compliance with select Articles of the upcoming EU AI Act, emphasizing radiology AI and potential differences in how this compliance is achieved. Greater regulatory clarity and clear guidelines are needed to guide providers and deployers in ensuring safe and fair patient care promoting fundamental rights.

## Data Availability

No datasets were generated or analyzed during the current study.
